# The Mouth–Mind Connection: Interplay of Oral and Mental Health in Older Adults

**DOI:** 10.3390/geriatrics11010008

**Published:** 2026-01-05

**Authors:** Alice Kit Ying Chan, Joanna Cheuk Yan Hui, Lindsey Lingxi Hu, Chun Hung Chu

**Affiliations:** Faculty of Dentistry, The University of Hong Kong, Hong Kong 999077, China; dralice@hku.hk (A.K.Y.C.); cyjaonna@hku.hk (J.C.Y.H.); hu_lingxi@connect.hku.hk (L.L.H.)

**Keywords:** older adults, elderly, oral health, prevention, caries, behavioural, aged, mental health

## Abstract

The global population aged 65 and older is expected to double from 761 million in 2021 to 1.6 billion by 2050. Despite often being treated separately in clinical practice and policy, oral health and mental health are fundamentally interconnected in older adulthood, forming a bidirectional relationship that exacerbates disability, social inequity, and systemic healthcare challenges. This narrative review aims to summarize the two-way relationship between mental and oral health and emphasize their combined impact on systemic health, social engagement, and independence among ageing populations. The bidirectional relationship has profound clinical significance. Untreated oral diseases induce chronic pain and cause social embarrassment, aggravating pre-existing depression and anxiety. Periodontal disease can worsen systemic conditions such as diabetes, cardiovascular disease, and dementia via a shared inflammatory pathway. Conversely, mental health issues—including depression, anxiety, cognitive decline, and the use of psychotropic medications—reduce motivation for oral care, prompt dental neglect, and affect salivary function, deteriorating oral health. Despite clear connections, systemic gaps persist, including fragmented healthcare systems, financial barriers, stigma, lack of awareness, and caregiver burnout. To address these challenges, strategies such as developing integrated care models to unify dental and mental health services, reforming policies to prioritize oral and mental health parity, advocating anti-stigma campaigns to clear the misconceptions, and implementing community-based healthcare programmes to reach underserved older adults are essential. By recognizing oral health as a vital component of mental resilience, societies can transform ageing into an era of empowered well-being, where the mouth–mind connection promotes holistic health rather than functional decline.

## 1. Introduction

The global demographic landscape is undergoing an unprecedented transformation, with the population aged 65 or older projected to double from 761 million in 2021 to 1.6 billion by 2050, representing nearly 17% of humanity [1]. This seismic shift, driven by declining fertility rates and advances in healthcare, underscores the urgent need to address age-related health challenges holistically. Among these, the interplay between mental health and oral health in older adults remains critically understudied, despite its profound implications for quality of life, morbidity, and healthcare costs [2]. Oral health and mental health, often siloed in clinical practice and policy, are inextricably linked in older adulthood, creating a bidirectional relationship that exacerbates disability, social inequity, and systemic strain.

Oral diseases are among the most prevalent non-communicable diseases globally [3]. Half of older adults have untreated caries, while 70% of them suffer from periodontal disease [4,5]. Substantial evidence has shown that periodontal diseases, a chronic localized inflammatory oral condition, are linked to several systemic illnesses like diabetes, cardiovascular disease, and dementia [6]. The endpoint of unattended dental caries and periodontal diseases is tooth loss. Edentulism, which affects almost one-quarter of the global older adult population, impairs chewing function, contributing to malnutrition—a risk factor for cognitive decline [7,8]. The World Health Organization (WHO) stated that those with the greatest need, such as the low-income population, people with disabilities, or older adults, often have the least access to oral healthcare services [9].

Concurrently, one in seven older adults aged 70 or over worldwide are living with mental disorders, with depression and anxiety being the most prevalent mental health conditions [10]. Late-life depression is often underdiagnosed due to somatic symptom overlap, such as fatigue, distress, and stigma [11]. Depression affects mood, concentration, motivation, sleep, and appetite, leading to behavioural changes [12]. Dementia, projected to afflict 152 million people by 2050, devastates individuals’ cognitive and functional capacities, complicating their self-care abilities like bathing, brushing teeth, and eating [12,13]. Critically, mental health conditions of older adults intersect with socioeconomic determinants such as isolation, poverty, and limited healthcare access, amplifying their vulnerability to physical decline [10].

Recent studies found that these two seemingly unrelated common geriatric comorbidities are, in fact, correlated [2,14]. The FDI World Dental Federation used “A happy mouth is a happy mind” as the theme of the World Oral Health Day 2025 to emphasise the mouth–mind connection [15]. Mental disorders negatively affect motivation and cognition in oral hygiene practice and reduce tolerance to dental treatment; oral diseases induce pain and limit function, impairing social interaction and self-esteem. Despite these connections, the oral health and mental health of older adults are addressed separately. Mental health services, scarcely involved in geriatric care, rarely screen for oral health issues [11]; dental professionals often lack training to address anxiety or cognitive impairment [16]. With the growing ageing population, it is paramount to bridge the gap between oral and mental healthcare to mitigate suffering, improve overall well-being, and honour the dignity of older adults.

The aim of this narrative review is to summarize the two-way relationship between oral and mental health, underscore their collective impacts on the ageing population, and discuss the barriers and facilitators for an integrated geriatric care model in addressing both oral and mental health to improve systemic health, dignity, and independence in ageing populations.

## 2. Methods

This review is based on English publications, including clinical studies and reviews identified through PubMed and Google Scholar, together with information obtained from websites such as those of the World Health Organization and FDI World Dental Federation. The last search date was 31 December 2025. The search keywords included “mental health”, “mental disorders”, “oral health”, “oral diseases”, “older adults”, and “elderly”.

## 3. Bidirectional Relationship Between Mental Health and Oral Health

The bidirectional relationship between mental health and oral health in older adults holds profound clinical significance, demanding urgent attention in geriatric care. Depression and anxiety diminish motivation for self-care, leading to infrequent brushing and the avoidance of dental visits, and increase the reliance on sugary diets for comfort [17,18]. People with dementia may forget to brush their teeth and have difficulties in performing proper daily oral hygiene practice or cooperating with dental procedures [12]. Antidepressants, antipsychotics, and antidementia medications, commonly prescribed to older adults, reduce salivary flow, exacerbating the risk of dental caries, periodontal disease, and oral infection [19].

**Figure 1:**
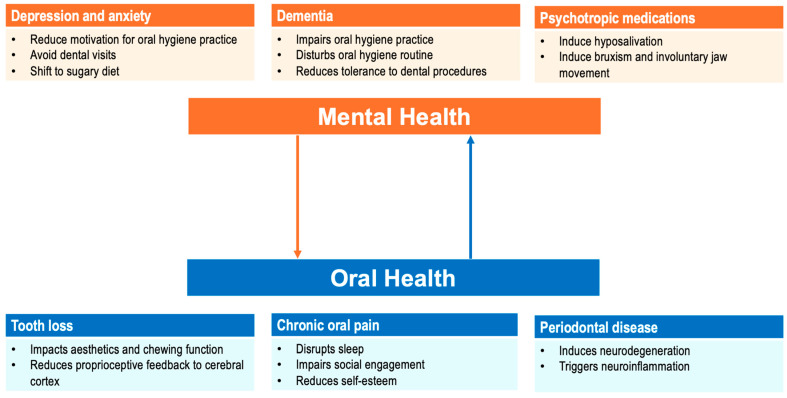
The bidirectional relationship between mental health and oral health. (Created with PowerPoint Version 16.101.3).

Conversely, poor oral health imposes psychological burdens: chronic pain from untreated oral diseases disrupts sleep and social engagement, fuelling depressive symptoms [20]. Tooth loss carries stigma, affects aesthetics, erodes self-esteem, and prompts social withdrawal—a precursor to loneliness, which increases the risk of depression by 28% [17]. Multiple tooth loss reduces chewing function and decreases prefrontal activations and cerebral blood flow, contributing to dementia. Periodontal disease triggers inflammatory cascades and induces neuroinflammation, which is implicated in Alzheimer’s disease [21,22]. Figure 1 summarizes the bidirectional relationship between mental and oral health.

## 4. Impact of Mental Health on Oral Health

The impact of mental health on oral health can come from (a) depression and anxiety, (b) cognitive decline and dementia, and (c) psychotropic medications.

### 4.1. Depression and Anxiety

Depression exerts a profound toll on oral health through behavioural and physiological pathways. The lethargy, anhedonia, and diminished self-worth characteristic of depression often manifest as a neglect of basic self-care [17]. Older adults with depression are more likely to skip tooth brushing twice daily and less likely to utilize oral health services for regular routine dental care compared to their non-depressed peers [23]. This neglect creates a breeding ground for plaque accumulation, leading to dental caries and periodontal diseases. Individuals with mental health conditions such as depression have sugar cravings to regulate their negative emotions. This poor dietary habit increases the risk of dental caries [24]. Anxiety disorders, particularly dental phobia, further compound these risks. One of the reasons for older adults avoiding dental care is fear rooted in traumatic past experiences or anticipatory pain [25]. This avoidance allows minor issues like initial carious lesions or gingival inflammation to escalate into dental abscesses, mobile teeth, or tooth loss [26]. The psychosocial consequences are equally dire: tooth loss correlates with social withdrawal, as almost 60% of edentulous older adults report difficulties in accepting tooth loss and hence may avoid social interactions due to embarrassment, deepening depressive symptoms in a cyclical manner [27].

### 4.2. Cognitive Decline and Dementia

Cognitive impairment, including Alzheimer’s disease and vascular dementia, disrupts the neural pathways required for routine oral hygiene. In early-stage dementia, individuals may forget to brush their teeth or misuse oral hygiene tools, while advanced stages render them dependent on caregivers for mouth care—a task many caregivers find challenging due to patient resistance or lack of professional training [28,29]. A 2024 qualitative survey of dementia caregivers revealed that most struggled with providing daily oral hygiene assistance, as dementia patients are often reluctant to perform or receive oral care [30]. Older adults with dementia do not understand why they need to open their mouth or why they need to brush their teeth [31]. These resistant behaviours result in painful conditions like dental caries and periodontal disease, which are frequently underreported due to communication barriers in dementia patients [28]. Older adults with late-stage dementia also show resistance to routine dental procedures, making preventive oral care challenging [28].

### 4.3. Psychotropic Medications

Studies showed that the use of psychotropic drugs increases with age and is up to 60–90% among frail older adults [32,33]. The widespread use of psychotropic medications, e.g., antidepressants or neuroleptics for depression and benzodiazepines or Z-drugs for sleep disorders in older adults, introduces iatrogenic risks to oral health [32]. Antidepressants, particularly tricyclics (e.g., amitriptyline), selective serotonin reuptake inhibitors (e.g., sertraline), and acetylcholine receptor blockers, reduce salivary flow by 35–50% [34]. Antipsychotics like risperidone, used in around one-fourth of older adults with dementia-related agitation, also induce hyposalivation [35]. Saliva plays an important role in oral health by maintaining a neutral pH oral environment, providing a reservoir of calcium and phosphate ions for remineralization, and containing a series of immunoglobulins for defence [19]. Drug-induced hyposalivation not only causes oral mucosal soreness and dysgeusia but also increases the risk of dental caries, periodontal diseases, and oral infections like oral candidiasis [36]. Compounding the issue, many psychotropic drugs also cause bruxism or tardive dyskinesia (involuntary jaw movements), leading to enamel fractures and temporomandibular joint disorders [37].

## 5. Impact of Oral Health on Mental Health

Periodontal disease, dental caries, and tooth loss, the common oral conditions in older adults, negatively affect mental health through (a) pain and functional limitations, (b) social stigma and isolation, and (c) systemic health connections.

### 5.1. Pain and Functional Limitations

Chronic oral pain, often stemming from untreated dental caries, advanced periodontal disease, or ill-fitting dentures, is a pervasive issue among older adults, with studies indicating that around 15% of older adults experience persistent oral discomfort [38,39]. Chronic oral pain disrupts sleep architecture by reducing restorative rapid eye movement sleep and increasing nighttime awakenings and is strongly correlated with heightened anxiety and depression [40]. For instance, a 2023 study found that older adults with toothache over the past 6 months were 1.5 times more likely to have depression than their pain-free peers [41].

Advanced dental caries and periodontal diseases, if left untreated, lead to tooth loss and hence limit functions such as chewing and swallowing [7]. Tooth loss or ill-fitting dentures negatively affect food choice and intake; older adults may shift to a softer diet with less fibre or meat, leading to deficiencies of micronutrients such as zinc, vitamin B12, and vitamin D—which are critical for cognitive function and mood regulation [7,42]. Studies found that vitamin B12 deficiency is a common cause of neuropsychiatric symptoms in the older population and is prevalent among older adults, affecting 10–15% of Western older adults [43,44]. Besides impairing nutritional intake, studies also suggested that tooth loss reduces chewing function and somatosensory feedback from the oral cavity to the central nervous system, leading to cognitive decline [21].

### 5.2. Social Stigma and Isolation

The psychosocial toll of poor oral health is profound, with tooth loss and visible dental caries carrying societal stigma that equates oral neglect with personal failure [45]. Tooth loss and poor oral conditions have a significant impact on aesthetics that may lead to social embarrassment and withdrawal [46]. Cultural perceptions amplify this stigma: in societies prioritizing youth and aesthetics, tooth loss and poor oral health are often misconstrued as a marker of ageing or low socioeconomic status, deepening shame. A cross-sectional study revealed that older adults with tooth loss were 65% more likely to have low subjective well-being, contributing to social withdrawal [45]. Social isolation is a potent risk factor for depression. Longitudinal data show that severely socially withdrawn older adults who have minimal family and social contact face a 50% higher risk of depressive symptoms [47]. Improving oral health can reverse these effects. A 2020 longitudinal study found that older adults receiving removable partial dentures for tooth replacement reported a 73% improvement in self-confidence within three months, underscoring oral health’s role in psychological resilience [48].

### 5.3. Systemic Health Connections

The mouth–body–mind connection is mediated by chronic inflammation and microbial dysbiosis. Periodontal pathogens like *Porphyromonas gingivalis* release pro-inflammatory cytokines, e.g., C-reactive protein, serum interleukin-6, and tumour necrosis factor-α, which enter systemic circulation, exacerbating systemic conditions such as atherosclerosis and insulin resistance [49,50]. These chronic medical conditions combined with periodontal diseases, in turn, elevate depression risk. Diabetic patients with periodontal disease are almost 30% more likely to develop depression and 50% more likely to develop anxiety than those without [51]. Moreover, stress-induced hormones related to depression increased the proliferation and growth of the periodontal pathogens *Tannerella forsythia* and *Fusobacterium nucleatum*, whereas the periodontal pathogen *Fusobacterium nucleatum* was associated with elevated corticosterone levels [52]. A minimally invasive approach with the use of probiotics, parabiotics, postbiotics, and ozonated substances may be considered to reduce the risk of dysbiosis and hence improve systemic health [53]. Alarmingly, periodontal disease is linked to neurodegenerative diseases. Amyloid-beta plaques in Alzheimer’s patients often contain oral bacteria, suggesting a direct pathway from periodontal disease to cognitive decline [22]. A systematic review concluded that improving oral hygiene care may play a positive role in maintaining the cognitive function of individuals with dementia [54]. Addressing oral health thus represents a cost-effective strategy to disrupt the vicious cycle linking oral, physical, and mental decline.

## 6. Barriers to Integrated Care

**Figure 2:**
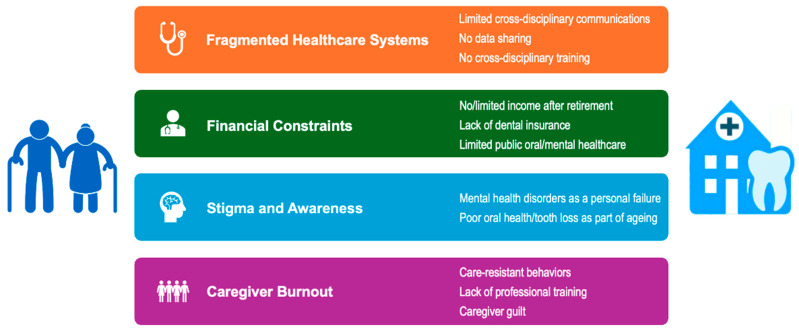
Barriers to integrated care. (Created with PowerPoint Version 16.101.3 and BioRender.com, accessed on 16 November 2025).

Despite clear connections between oral and mental health, systemic gaps in addressing the issue include (a) fragmented healthcare systems, (b) financial constraints, (c) stigma and awareness, and (d) caregiver burnout (Figure 2).

### 6.1. Fragmented Healthcare Systems

The separation of dental care from mainstream medical healthcare systems represents a critical barrier to holistic geriatric care [55]. In most countries, these dental and medical disciplines operate in siloed frameworks, with distinct insurance structures, training protocols, and clinical workflows [55]. For example, in the US, Medicare excludes routine dental care, whereas mental health services are covered only under specific conditions, creating bureaucratic hurdles for integrated treatment [56,57]. A 2021 survey found that only 15% of primary care providers had team experience with oral health professionals [58]. This fragmentation delays diagnoses: depression-induced xerostomia may go unnoticed by psychiatrists, whereas dentists might overlook signs of anxiety in patients avoiding treatment. Electronic health records which rarely include dental data and limited communication between oral and other healthcare providers stall interdisciplinary collaboration [55]. Moreover, cross-disciplinary training is absent in most medical curricula [55]. For instance, a 2019 survey of American geriatrician fellows revealed that almost one-quarter of them did not receive any non-clinical instruction on oral health and over 80% of them spent zero hours in a dental setting with a dental professional over the whole course of their training [59]. Until policies mandate interoperable systems and incentivize collaborations, older adults will continue to fall through the gaps of a fractured system.

### 6.2. Financial Constraints

Financial barriers disproportionately harm older adults, particularly in nations without universal dental coverage [60]. In the US, 70% of older adults lack dental insurance after retirement, as employer-based plans typically end at 65, and Medicare offers limited coverage—only 2% of beneficiaries utilize its sparse dental benefits. With dental insurance coverage caps from USD 1000 to USD 1500, out-of-pocket costs are prohibitive [61]. Consequently, 60% of low-income older adults forgo dental care annually [62]. This cost-driven neglect has dire consequences: untreated dental caries and periodontal disease increase the risk of cardiovascular diseases, diabetes, aspiration pneumonia, and dementia, which incur far higher hospitalization costs [63]. Conversely, Germany’s universal coverage model reduces the prevalence of edentulism to 5% among older adults, compared to 13% in the US [64,65]. Without systemic reforms to address affordability, oral and mental health disparities will widen, perpetuating cycles of poverty and illness.

### 6.3. Stigma and Awareness

Cultural stigma and misconceptions about ageing further marginalize oral and mental health needs. Mental health stigma remains pervasive: many older adults view depression as a “personal failure” rather than a medical condition, leading to underreporting or avoidance of treatment [66]. A 2022 survey found that although over 80% of older adults with depression perceived access to depression care in the universal healthcare system as convenient, less than 30% of them sought professional help [67]. Older adults declined mental healthcare often due to embarrassment, fear of judgment, or disbelief in treatment [68]. Similarly, many older adults often regard poor oral health or tooth loss as a “normal” process of ageing and do not perceive a need for dental care [16,69]. Educational gaps compound the issue: only 30% of older adults recognize that periodontal disease is linked to diabetes, and many older adults think that oral health is not part of physical health [16,70]. Training primary healthcare providers to initiate conversations about oral and mental health—and normalizing these topics in elderly health centres—could dismantle stigma and empower early intervention.

### 6.4. Caregiver Burnout

Caregivers face immense challenges in delivering oral care to cognitively impaired older adults [71]. Care-resistant behaviours, common in mid-stage dementia, often lead to confrontations during oral hygiene practice: 80% of certified nursing assistants reported experiencing struggles during mouth care, with behaviours ranging from mild resistance like refusing mouth opening to extreme resistance like hitting or kicking [72]. The emotional toll is profound: 65% of dementia caregivers reported significant guilt when they became angry in response to resistant behaviour or when they felt they were not providing proper care. Guilt is one of the diagnostic criteria for major depressive disorder, and almost 40% of dementia caregivers develop clinical depression [73]. However, structural support is minimal, with many caregivers lacking professional training in oral care. Almost 50% of caregivers feel that they do not spend the right amount of time on oral hygiene practice for their care recipients, and 20% of them doubt if they are providing them with proper oral hygiene practice [71]. Professional oral care training for caregivers is crucial to relieve their burnout and preserve their well-being.

## 7. Strategies for Holistic Intervention

These interconnected barriers demand comprehensive and policy-driven solutions. Integrating oral and mental healthcare services, implementing universal health coverage, and launching anti-stigma campaigns and community-based healthcare programmes are the critical first steps. By prioritizing older adults’ holistic well-being, societies can transform ageing from a narrative of decline to one of resilience and dignity.

### 7.1. Integrated Care Models

Integrated care models bridge the divide between oral and mental health services through interdisciplinary collaborations. Several programmes provide integrated care models in the US. Among these, the Health Resources and Service Administration Health Centre Program integrates medical, dental, and behavioural healthcare services to address the systemic, mental, and oral health of the population [74]. Patients who received these integrated healthcare services showed fewer hospitalizations and emergency visits. Moreover, they had increased access to preventive healthcare services compared to those attending other primary care settings. Medicaid, who funded these healthcare services, found that patients in this programme had 24% lower healthcare spending [74]. The integrated care model can control the shared risk factors simultaneously, assist early detection of oral and mental health problems, aid health surveillance and data sharing, and allow better allocation of resources and manpower [55].

Additionally, caregivers of older adults with dementia should receive integrated training to take care of care recipients’ oral and general health. For instance, the MOTIVATE at Home Program is a free oral health education programme designed for informal caregivers, e.g., family members or friends of older adults who reside at home [75]. It aims to improve oral health literacy and teach practical, evidence-based home oral healthcare. It also educates caregivers about oral–systemic connections and communication techniques for discussing care recipients’ oral health with other healthcare providers [75].

### 7.2. Policy Reforms

Achieving systemic change requires policy reforms that ensure equitable coverage for both oral and mental health. The FDI World Dental Federation advocates for the inclusion of oral health as part of universal health coverage [60]. The WHO also supports the inclusion of mental healthcare within universal health coverage in the WHO Special Initiative for Mental Health (2019–2023): all people achieve the highest standard of mental health and well-being [76]. With the inclusion of oral and mental healthcare in universal health coverage, older adults can receive integrated care without financial hardship [77]. Oral and mental healthcare can be more affordable, accessible, and available [60,76]. Japan has implemented universal oral health coverage for over a decade, and the percentage of older adults aged 80 or above with 20 natural teeth or more (functional dentition) increased from 7% in 1989 to more than 50% in 2016 [60].

### 7.3. Anti-Stigma Campaigns

Regarding the serious global burden of mental health problems and widespread public misconceptions about these conditions, the WHO has launched the Comprehensive Mental Action Plan 2013–2030 to fight against misconceptions, stigma, and discrimination around mental health conditions. A series of testimonial videos of people with lived experience of mental health conditions has been shared with the public to enhance mental health literacy [78].

Additionally, the WHO has prepared a testimonial video of an older Japanese adult with complete dentition, who shared his story of how oral health ensures healthy ageing by enabling enjoyment of food and social life. It helped to clear the misconception that tooth loss is an inevitable process of ageing [79].

### 7.4. Community-Based Healthcare Programmes

**Figure 3:**
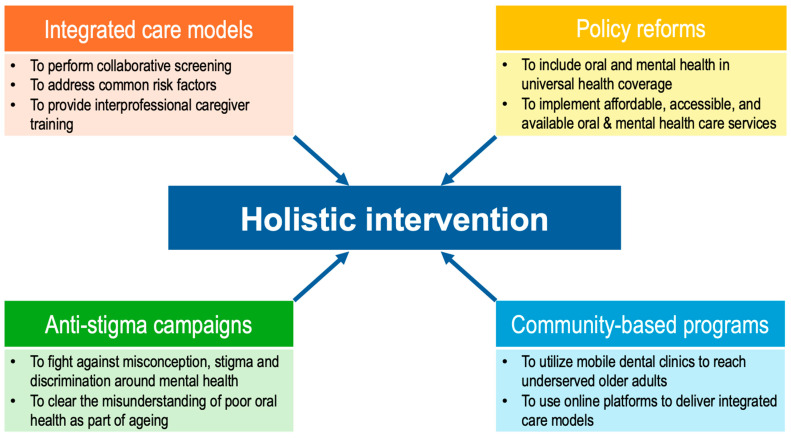
Strategies for Holistic Intervention. (Created with PowerPoint Version 16.101.3).

Community initiatives are pivotal for reaching underserved older adults, particularly in rural or low-income areas. In the US, Columbia University coordinates the ElderSmile programme with Senior Centres to offer a community-based integrated healthcare programme for older adults in impoverished communities. The staff and students from the College of Dental Medicine of Columbia University provide oral care in terms of prevention, education, screening, and treatment services through mobile van visits. Additionally, they work together with Senior Centres to offer integrated care including diabetes and hypertension screening to promote older adults’ oral and general health [80]. The Program to Encourage Active, Rewarding Lives (PEARLS) is an evidence-based programme designed to reduce depression symptoms in older adults and improve their quality of life through self-management skills. This programme uses a home-based/community-based collaborative care model to teach problem-solving, social, and physical activity planning skills for self-management. They offer in-person or video conference counselling for remote older adults. Almost half of the participants showed at least 50% reduction in depressive symptoms after the programme [81]. Figure 3 summarizes the strategies for Holistic Intervention. By prioritizing collaboration, equity, and prevention, healthcare systems can foster resilience and dignity in ageing populations.

This narrative review provides a broad overview of the interplay between oral health and mental health in older adults. However, the current evidence is mainly based on cross-sectional studies. Future research must prioritize longitudinal studies to provide a clear direction of the relationship. Additionally, research to quantify the lifespan benefits of interventions like co-located dental–mental health clinics and community healthcare programmes, particularly in marginalized communities, should be conducted.

## 8. Conclusions

The profound interplay between mental and oral health in older adults demands urgent recognition as a public health priority, given its cascading impacts on morbidity, mortality, and societal costs. Addressing systemic barriers—such as fragmented care systems, financial inequities, cultural stigma, and caregiver burnout—through integrated care models, policy reforms, anti-stigma campaigns, and community-driven initiatives can reduce preventable suffering and hospitalizations while restoring dignity and social engagement. By reframing oral health as a vital component of mental resilience, societies can transform ageing into an era of empowered well-being, where the mouth–mind connection fuels holistic health rather than compounding decline.

## Data Availability

No new data were created or analyzed in this study.
